# Prevalence of *Helicobacter pylori *infection and associated factors among adults in Southern Brazil: a population-based cross-sectional study

**DOI:** 10.1186/1471-2458-5-118

**Published:** 2005-11-10

**Authors:** Ina S Santos, Jose Boccio, Ari S Santos, Neiva CJ Valle, Camila S Halal, Marta Colvara Bachilli, Ricardo D Lopes

**Affiliations:** 1 Department of Social Medicine, Faculty of Medicine, Federal University of Pelotas, Pelotas, Brazil; 2 Laboratory of Stable Isotopes applied to Medicine and Biology, School of Pharmacy and Biochemistry, University of Buenos Aires, Buenos Aires, Argentina; 3 Department of Analytic and Inorganic Chemistry, Instituto f Chemistry and Geosciences, Federal University of Pelotas, Pelotas, Brazil

## Abstract

**Background:**

*Helicobacter pylori (Hp) *infection is associated with several upper gastrointestinal disorders. Local data on the epidemiology of the infection are scarce in Brazil. The purpose of this study is to measure the prevalence rate and to explore the associated factors among the adult population living in Pelotas, a southern Brazilin city.

**Methods:**

This was a population-based cross-sectional study. Through a multi-stage sampling method all individuals 20 years and over living at the selected households at the urban area of the city were interviewed regarding past and current socio-economic indicators; demographic characteristics; nutritional and behavioural habits; and history of upper gastrointestinal symptoms.*Hp *infection was ascertained through the ^13^C-UBT. Due to the high prevalence, data were analysed through robust Poisson regression. All analyses took into account the family clustering of the data.

**Results:**

Among 563 eligible individuals, 363 agreed to perform the ^13^C-UBT (refusal rate of 35.5%). Refusals were associated with female sex, consumption of mate drinking, and presence of upper gastrointestinal symptoms. The prevalence rate of *H. pylori *infection was 63.4% (95%CI 59.3%–69.3%). In crude analyses, prevalence was associated with increasing age, non-white skin colour, lower current family income, lower education level, higher size of the family, low socio-economic conditions in childhood, higher number of siblings and attendance to day-care centres in childhood, and presence of dyspeptic symptoms. In adjusted analysis the level of education of the father was inversely associated with the infection, whereas number of siblings and attendance to day-care centre in childhood were directly associated with it. Non-white skin colour remained significantly associated with increased prevalence even after allowing for past and current socio-economic characteristics, age and sex. Compared to non-symptomatic individuals, those reporting dyspeptic symptoms presented a higher prevalence of the infection even after allowing for current and past socio-economic conditions, ethnicity, age, and sex.

**Conclusion:**

*Hp *infection is as common among adults in southern Brazil as it is in other developing countries. Socio-economic conditions in childhood besides ethnicity and presence of dyspeptic symptoms were the factors significantly associated with the infection.

## Background

*Helicobacter pylori (H. pylori) *infection is chronic and common throughout the world, with a higher prevalence in developing than in developed countries [1]. *H. pylori *infection causes gastritis and is the most important risk factor for peptic ulcer disease (gastric and duodenal)[2,3]. It also contributes to the onset of gastric cancer and primary gastric B-cell lymphoma [2,4] and more recently has been investigated as a risk factor for ischemic heart disease[5] and intrauterine growth restriction[6,7].

In Brazil, in 1998, among the malignancies, gastric cancer was second only to lung cancer as the cause of mortality in men[8]. Despite of this, local epidemiological data on *H. pylori *infection are scarce. Through a non-restrictive search at Medline and SciElo databases using "*Helicobacter pylori" *and "prevalence or seropositivity" and "Brazil or Brazilian" as title/abstract descriptor terms, only seven studies primarily planned to measure prevalence of the infection among adult non-patient individuals were found [9-15]. Five of these studies had been conducted among selective populations (abattoir workers[9], Japanese Brazilians and Japanese residents in Brazil[11,12], blood donors[14], and native populations from Brazilian Western Amazon)[15]. Overall prevalence rate in those studies varied from 48% among Japanese Brazilians living in four different cities in Brazil[12] to 84.7% among the adult residents of a rural area of the state of Mato Grosso[10].

This paper reports the prevalence rate of the *H. pylori *infection and the factors that showed to be associated with the infection in a study conducted in Pelotas, a city of 320,000 inhabitants, located in the state of Rio Grande do Sul (southern Brazil).

## Methods

This was a population-based cross-sectional study. A multi-stage sampling method, taking the structure of the population as a basis, was used for sample selection. Fifty eight of the 404 census tracts of the city were randomly chosen and, subsequently, a random sample of five households per census tract was selected to the study. All adults (20 years and over) living in the selected households were invited to participate. Unoccupied houses were excluded and the house next-door was selected. In the case of temporary absence of one or more adults from the household (students, workers) the interviewer returned later, or during the next day, to perform the interview. After three unsuccessful attempts of contact, at different days, the adult/household was considered a loss. Refusals were also considered losses.

Subjects were interviewed at home by trained field workers using a standard questionnaire. Information on current (family monthly income in minimum wages, educational level, marital status, and size of the family) and past socio-economic characteristics (history of having lived only in urban or in urban and rural areas, level of formal education of the parents of the interviewee, number of siblings, and attendance to day-care centres in childhood) was collected.

Age was gathered in complete years. Gender and ethnicity (as defined according to skin colour) were observed by the interviewer. History of upper abdominal complaints in the last year: dyspeptic symptoms (pain or discomfort), substernal burning pain, decreased appetite, abdominal fullness, abdominal bloating, early satiety, and vomiting were investigated. History of peptic ulcer disease and gastric cancer in first degree relatives was explored.

Frequency of consumption of raw vegetables, coffee, alcoholic beverages and mate (*Ilex paraguayensis*) were collected. Smoking history was also provided by the interview. Changes in nutritional habits, alcoholic beverages ingestion and in smoking pattern due to the presence of upper abdominal symptoms were also investigated.

*H. pylori *infection was assessed by the isotope technique, the ^13^C-Urea Breath Test (^13^C-UBT) after at least six hours fastening. Each dose consisted into about 50 gram portion of ^13^C-urea (Cambridge Isotope Laboratories Inc., Massachusetts, USA). The samples were measured in a mass spectrometer coupled to a gas chromatographer (FinniganMAT GmbH, ThermoQuest Corp., Bremen, Germany). Subjects with an excess δ^13^CO_2 _value of > 3.5 per ml were defined as *H. pylori *positive[16].

Due to the high prevalence of the *H. pylori *infection, prevalence rates, prevalence ratios, and the respective 95% confidence intervals were calculated through robust Poisson regression[17], taking the family clustering of the data into account. The Stata 8.0 package was used for these analyses. A hierarchical model of analysis[18] was applied according to a conceptual framework established *a priori*, through which caudal variables were adjusted for cranial ones. At the first most cranial level were the socio-economic characteristics in childhood. At the second level, the current socio-economic variables, followed at the third level by the demographic factors. In the fourth and fifth levels, lifestyle characteristics and complaints of upper gastrointestinal symptoms, respectively, were entered to the model. Variables significant at their level were kept in the model even whether the inclusion of caudal variables turned their association with the outcome statistically non significant. At every level, a backward selection of variables was performed. Those presenting a p value ≤ 0.20 were kept in the model for adjustment purpose. Only variables with p value < 0.05 were considered significantly associated with *H. pylori *infection.

The study protocol was cleared by the Ethical Committee of the Federal University of Pelotas. A written informed consent was obtained from participants before enrolment in the study.

## Results

### Test compliers *versus *non-compliers

A total of 277 households were visited. In all, 563 adults answered the questionnaire and of them 363 accepted to have the ^13^C-UBT performed, a refusal rate of 35.5%. Tables 1, 2, 3, 4 describe compliers and non-compliers according to the independent variables investigated. Compliance to the test was significantly (p < 0.05) associated with sex (higher among the women), mate intake (higher among the consumers), and presence of most of the gastrointestinal symptoms explored (higher among the symptomatic individuals). The statistical significance of the association between compliance and current family income was borderline (p = 0.09).

**Table 1:** Demographic and socio-economic characteristics of non-complier and complier subjects, and prevalence of *H. pylori *infection

**Characteristics**	**Test non-compliers (n = 200) n (%)**	**Test compliers (n = 363) n (%)**	**p**^1^	**Prevalence**^2 ^**n (%)**	**p**^3^
**Age (years)**			0.6		0.02^4^
20–29	53 (26.5)	84 (23.4)		40 (47.6)	
30–39	35 (17.5)	78 (21.7)		56 (71.8)	
40–49	42 (21.0)	80 (22.3)		57 (71.3)	
≥ 50	70 (35.0)	117 (32.6)		78 (66.7)	
**Sex**			0.009		0.1
Male	95 (47.5)	129 (35.9)		76 (58.9)	
Female	105 (52.5)	230 (64.1)		155 (67.4)	
**Ethnicity**			0.6		<0.001
White	151 (75.5)	279 (77.7)		165 (59.1)	
Non white	49 (24.5)	80 (22.3)		66 (82.5)	
**Family income (MW)**			0.09		<0.001^4^
≤ 1.00	27 (14.1)	37 (10,5)		31 (83.8)	
1.01–3.00	66 (34.4)	138 (39.0)		92 (66.7)	
3.01–5.00	42 (21.9)	99 (28.0)		71 (71.7)	
≥ 5.01	57 (29.7)	80 (22.6)		35 (43.8)	
**Education**			0.5		0.001^4^
≤ 4	39 (21.5)	69 (19,2)		49 (71.0)	
5–7	41 (22.7)	84 (23.4)		60 (71.4)	
8–10	32 (17.7)	72 (20.1)		48 (66.7)	
≥ 11	69 (38.1)	109 (30.4)		53 (48.6)	
**Marital status**			0.4		0.8
With partner	127 (63.5)	241 (67.1)		156 (64.7)	
Without partner	73 (36.5)	118 (32.9)		75 (63.6)	
**Size of present family**			0.7		0.03
1–3	92 (46.0)	171 (47.6)		100 (58.5)	
≥ 4	108 (54.0)	188 (52.4)		131 (69.7)	

^1^p of difference between test-compliers and test non-compliers according to the independent variables

^2 ^prevalence among test-compliers

^3^p of the association between the independent variable and the prevalence of *H. pylori *infection

^4^test for linear trend

**Table 2:** Past socio-economic among non-complier and complier subjects, and prevalence of *H. pylori *infection.

**Characteristics**	**Test non-compliers (n = 200) n (%)**	**Test compliers (n = 363) n (%)**	**p**^1^	**Prevalence**^2 ^**n (%)**	**p**^3^
**Place of living**			0.1		0.2
Urban only	133 (66.5)	217 (60,4)		135 (62.2)	
Urban and rural	66 (33.5)	142 (39.6)		96 (67.6)	
**Mother education**			0.6		0.001^4^
≤ 4	108 (63.9)	203 (65.1)		143 (70.4)	
5–7	29 (17.2)	61 (19.6)		38 (62.3)	
8–10	13 (7.7)	16 (5.1)		8 (50.0)	
≥ 11	19 (11.2)	32 (10.3)		12 (37.5)	
**Father education (y)**			0.2		0.001^4^
≤ 4	101 (63.9)	181 (50.4)		127 (70.2)	
5–7	21 (13.3)	59 (16,4)		39 (66.1)	
8–10	12 (7.6)	24 (6.7)		11 (45.8)	
≥ 11	24 (15.2)	30 (8.4)		9 (30.0)	
**N**° **siblings**			0.9		0.001^4^
0–2	60 (30.2)	113 (31.5)		55 (48.7)	
3–4	49 (24.6)	77 (21.4)		54 (70.1)	
5–6	35 (17.6)	67 (18.7)		43 (64.2)	
≥ 7	55 (27.6)	102 (28.4)		79 (77.5)	
**Day-care center childhood**			0.2		<0.001
Yes	11 (5,5)	11 (3.1)		10 (90.9)	
No	188 (94.5)	348 (96.9)		221 (63.5)	

^1^p of difference between test-compliers and test non-compliers according to the independent variables

^2 ^prevalence among test-compliers

^3^p of the association between the independent variable and the prevalence of *H. pylori *infection

^4^test for linear trend

**Table 3:** Current lifestyle among non-complier and complier subjects, and prevalence of *H. pylori *infection.

**Characteristics**	**Test non-compliers (n = 200) n (%)**	**Test compliers (n = 363) n (%)**	**p**^1^	**Prevalence**^2 ^**n (%)**	**p**^3^
**Smoking**			0.6		0.3^4^
Never smoked	99 (49.7)	164 (45.7)		99 (60.4)	
Ex-smoker	44 (22.1)	88 (24.5)		63 (71.6)	
Current smoker	56 (28.1)	107 (29.8)		69 (64.5)	
**Alcohol intake**			0.9		0.07
No	101 (50.8)	186 (51.8)		128 (68.8)	
Yes	98 (49.2)	173 (48.2)		103 (59.5)	
**Coffee intake (d/wk)**			0.4		0.02^4^
0	27 (13.6)	38 (10.6)		18 (47.4)	
1–6	21 (10.6)	43 (12.0)		25 (58.1)	
7	151 (75.9)	278 (77.4)		188 (67.6)	
**Mate intake (d/wk)**			0.03		0.3^4^
0	70 (35.2)	81 (22.6)		58 (71.6)	
1–6	39 (19.6)	101 (28.2)		59 (58.4)	
7	90 (45.2)	176 (49.2)		113 (64.2)	
**Raw vegetables (d/wk)**			0.2		0.6^4^
0	34 (17.2)	51 (14.4)		34 (66.7)	
1–6	113 (57.1)	188 (53.1)		116 (61.7)	
7	51 (25.8)	115 (32.5)		78 (67.8)	

^1^p of difference between test-compliers and test non-compliers according to the independent variables

^2 ^prevalence among test-compliers

^3^p of the association between the independent variable and the prevalence of *H. pylori *infection

^4^test for linear trend

**Table 4:** Upper gastrointestinal symptoms among non-complier and complier subjects, and prevalence of *H. pylori *infection.

**Characteristics**	**Test non-compliers (n = 200) n (%)**	**Test Compliers (n = 363) n (%)**	**p**^1^	**Prevalence**^2 ^**n (%)**	**p**^3^
**Dyspeptic symptoms**			0.05		0.01
No	172 (86.4)	286 (79.7)		176 (61.5)	
Yes	27 (13.6)	73 (20.3)		55 (75.3)	
**Substernal burning**			0.01		0.09
No	108 (54.5)	155 (43.2)		92 (59.4)	
Yes	90 (45.5)	204 (56.8)		139 (68.1)	
**Decreased appetite**			0.001		0.2
No	164 (82.4)	246 (68.9)		154 (62.6)	
Yes	35 (17.6)	111 (31.1)		77 (69.4)	
**Abdominal fullness**			0.001		0.3
No	188 (94.5)	307 (85.5)		195 (63.5)	
Yes	11 (5.5)	52 (14.5)		36 (69.2)	
**Early satiety**			0.1		0.8
No	192 (96.5)	335 (93.3)		216 (64.5)	
Yes	7 (3.5)	24 (6.7)		15 (62.5)	
**Vomiting**			0.06		0.6
No	194 (97.5)	337 (93.9)		218 (64.7)	
Yes	5 (2.5)	22 (6.1)		13 (59.1)	
**Abdominal bloating**			0.03		0.5
No	172 (86.9)	285 (79.4)		181 (63.5)	
Yes	26 (13.1)	74 (20.6)		50 (67.6)	
**Family history peptic disease 1° degree relatives**			0.7		0.9
No	23 (43.4)	57 (40.7)		36 (63.2)	
Yes	30 (56.6)	83 (59.3)		53 (63.9)	
**Family history gastric cancer 1**°**degree relatives**			0.6		0.9
No	25 (83.3)	57 (68.7)		39 (68.4)	
Yes	5 (16.7)	26 (31.3)		18 (69.2)	

^1^p of difference between test-compliers and test non-compliers according to the independent variables

^2 ^prevalence among test-compliers

^3^p of the association between the independent variable and the prevalence of *H. pylori *infection

At the whole sample (n = 563) no statistical association between sex and mate drinking was detected: 73.1% and 72.8%, respectively, of the men and women were regular mate drinkers (at least once a week). The association between mate drinking and dyspeptic symptoms was in the limit of the statistical significance both in crude (p = 0.05) and in adjusted analysis (p = 0.06): 19.9% of the mate drinkers reported dyspeptic symptoms against 12.6% of the non-drinkers. The same was observed with the association between sex and dyspepsia (p = 0.9): the prevalence of dyspeptic symptoms was slightly higher among women (20.3%) than among men (14.3%). Stratified analysis according to the compliance status of the interviewees did not change these findings, except by the higher p-values.

### Description of the compliers

On Table 1, among the compliers, almost two thirds were women (64.1%) and lived with a partner (67.1%). More than 50% shared household with four or more relatives and 49.5% were from families earning no more than three Brazilian minimum wages per month. Almost one third of the compliers (30.4%) had more than ten years of formal education. Regarding past socio-economic characteristics (Table 2), 39.6% were born or spent part of their lives at the rural area. The level of education of the mother and the father for 65.1% and 50.4%, respectively, of the compliers was of four years or less. More than one fourth of the individuals (28.4%) belonged to families with seven or more siblings. A small proportion of the participants (3.1%) attended to day care centres in childhood.

Table 3 shows that 29.8% were smokers. Consumption of alcoholic beverages was reported by 48.2% of the interviewees. Daily coffee drinking was reported by 77.4% of the participants, while 49.2% informed to be daily mate drinkers. Consumption of raw vegetables at least once a week was reported by 85.6% of the individuals.

On Table 4, prevalence of dyspeptic symptoms (upper abdominal pain or discomfort in the last year) was of 20.3%. Other symptoms like substernal burning pain, decreased appetite, abdominal bloating, abdominal fullness, early satiety, and vomiting were complained in the last year by, respectively, 56.8%, 31.1%, 20.6%, 14.5%, 6.7%, and 6.1% of the interviewees. One hundred fifty seven individuals had sought medical care due to abdominal symptoms. Gastritis, peptic ulcer disease and gastro-oesophageal reflux were reported, respectively, by 38.9%, 9.3% and 7.4% of the 54 participants who were aware, at the moment of the interview, of the medical diagnosis to their symptoms.

### Crude prevalence rates and adjusted prevalence ratios for *H. pylori *infection

Only four of the 363 individuals who had the ^13^C-UBT had undetermined test result. Overall prevalence of *H. pylori *infection among the remaining 359 subjects was of 64.3% (95%CI 59.3%–69.3%). In crude analysis, prevalence of *H. pylori *infection was statistically associated with age, ethnicity, family monthly income, level of education, size of the actual family, and past socio-economic indicators (Tables 1 and 2). The prevalence increased with age although remaining relatively constant after the twenties: from 47.6% among individuals 20–29 years old to more than 70% among the 30–49 year old. Prevalence among non-white individuals was 40% higher than the observed among whites (respectively, 82.5% and 59.1%; p < 0.001). Infection rate among the poorest (83.8%) was almost twice as high as among those from families earning more than five minimum wages a month (43.8%). Association with educational level was reversed: the higher the education level the lower the prevalence of *H. pylori *infection (p = 0.001). Comparatively to individuals from smaller families (1–3 members), those living with four or more relatives had a prevalence rate of infection 19% higher (respectively, 58.5% and 69.7%). As with the participant level of education, association with both maternal and paternal education was reversed. Prevalence was 87% higher among those whose mother presented an educational level ≤ 4 years, comparatively to those whose mothers had ≥ 11 years of education (p = 0.001). The association with the paternal schoolarity was even stronger, with 2.3 fold increase in prevalence rate among individuals whose father had low schoolarity, comparatively to those with eleven years or more of formal education. A linear trend was observed between number of siblings in childhood and prevalence of the infection with higher prevalence according to the increase in the number of siblings. Prevalence was 43% higher among those who had attended day-care centres in childhood comparatively to those who did not (respectively, 90.9% and 63.5%).

Among lifestyle characteristics, only coffee intake was significantly associated with the infection. A linear trend was observed between weekly frequency of coffee consumption and the *H. pylori *infection rate (Table 3). Daily drinkers presented a prevalence rate of 67.6%, whereas among the non-drinkers the prevalence was 47.4%. The statistical significance of the association with alcohol consumption was borderline (p = 0.07), with non-consumers presenting a higher prevalence of infection than consumers (respectively, 68.8% and 59.5%).

On Table 4, only the presence of dyspeptic symptoms was significantly associated with the occurrence of *H. pylori *infection, with symptomatic individuals having a prevalence 22% higher than the observed among non-symptomatic ones (p = 0.01). The statistical significance of the association between substernal burning and the infection was borderline (p = 0.09): symptomatic individuals had a prevalence rate 15% higher than those not reporting this symptom. No association between family history of peptic ulcer disease or gastric cancer and *H. pylori *infection was detected.

Table 5 presents crude and adjusted prevalence ratios of the independent variables significantly associated with the outcome. Level of education of the father, number of siblings and attendance to day-care centres in childhood remained statistically significant after adjusting to all other past socio-economic indicators. Father education was protective against the occurrence of the infection. Subjects whose father had eleven years or more of formal education presented a 53% decrease in the probability of infection when compared to those whose father had ≤ 4 years of schoolarity. The occurrence of infection increased linearly with the number of siblings: individuals from larger families in childhood (≥ 7 siblings) presented a probability 55% higher of being *H. pylori *positive than those from families with no more than two children. Among those who attended day-care centres in childhood, the probability of the infection was 53% higher than among their counterparts.

**Table 5:** Crude and adjusted prevalence ratios of independent variables for *H. pylori *infection. (n = 359)

**Characteristics**	**Crude PR**	**95% CI**	**Adjusted PR**	**95% CI**	**p**
**Father education)**					0.01^1^
≤ 4	1.00		1.00		
5–7	0.94	0.77 – 1.16	0.98	0.80 – 1.20	
8–10	0.65	0.42 – 1.02	0.69	0.46 – 1.06	
≥ 11	0.42	0.24 – 0.74	0.47	0.28 – 0.79	
**N° siblings**					0.001^1^
0–2	1.00		1.00		
3–4	1.44	1.13 – 1.83	1.44	1.11 – 1.86	
5–6	1.32	1.02 – 1.71	1.27	0.96 – 1.68	
≥ 7	1.59	1.28 – 1.98	1.55	1.22 – 1.98	
**Day-care centre in childhood**					0.001
Yes	1.01	1.01 – 1.01	1.53	1.18 – 1.97	
No	1.00		1.00	1.00	
**Current family income (MW)**					0.07^1^
≤ 1.00	1.00		1.00		
1.01–3.00	0.80	0.66 – 0.96	0.86	0.69 – 1.06	
3.01–5.00	0.86	0.71 – 1.03	0.97	0.79 – 1.19	
≥ 5.01	0.52	0.39 – 0.69	0.68	0.49 – 0.93	
**Ethnicity**					<0.001
White	1.00		1.00		
Non white	1.39	1.21 – 1.61	1.32	1.14 – 1.54	
**Dyspeptic symptoms**					0.002
No	1.00		1.00		
Yes	1.22	1.04 – 1.44	1.28	1.10 – 1.50	

^1^test for linear trend

The present socio-economic variables significantly associated with *H. pylori *infection in crude analysis (current family income, education and size of the present family) lost their statistical significance after controlling for past socio-economic indicators. Family monthly income presented a borderline statistical significance (p = 0.07). Individuals from families earning five or more minimum wages presented a probability of infection 32% lower than those from the poorest families. However, the confidence limits of the adjusted prevalence ratios of all the other categories of the variable included the unit.

Among the demographic variables, after adjusting for past and present socio-economic factors, only ethnicity remained significantly associated with the outcome. The adjusted prevalence ratio for non-whites was 32% higher than that of the whites. Age which was linearly and significantly associated with the infection in crude analysis lost its significance after adjusting for confounders (p = 0.1). Together with sex (p = 0.1) the variable age was kept in the model for adjustment of the subsequent variables.

None of the lifestyle characteristics were significantly associated with *H. pylori *infection in adjusted analysis. Among the variables of this group, only weekly frequency of coffee intake (p = 0.1) was kept in the model for controlling the upper gastrointestinal symptoms. Individuals complaining of dyspeptic symptoms in the last year had an adjusted prevalence ratio 28% higher than their counterparts. All other symptoms investigated failed to show statistically significant association with the outcome.

Effect modification analyses were conducted to investigate the possible interaction between age and presence of dyspeptic symptoms, age and father education, age and family income, and age and ethnicity over *H. pylori *prevalence. None of those joint effects showed to be statistically significant in crude or adjusted analyses.

## Discussion

The principal limitation of this study is the high refusal rate of the eligible individuals to realization of the ^13^C-UBT. Despite the simplicity and the non-invasiveness character of the ^13^C-UBT, which could make it suitable for use in field-based population studies, the refusal rate was very high (36.2%) what, as will be considered in detail below, can compromise the validity of some of the observed results. The need of 6-hour fastening is probably one of the main constraints for its use in field-based surveys. Nevertheless, similar or even higher refusal rates were reported by other authors using different methods in population-based studies collecting primary data: 32% in Australia[19], 41.6% in Northern Ireland[20] and 74% in England[21]. Comprehensively, normal individuals are less compelled to adhere to a test than patients attending consultation for abdominal symptoms, the target population more frequently found in the published literature on *H. pylori *prevalence. Studies conducted among symptomatic individuals, however, suffer from a different kind of selection bias since findings obtained from samples of gastroenterological patients are not necessarily representative of the whole population and can not be extrapolated with confidence to non-patient individuals. It is not surprising that prevalence rates available in the literature come mainly from the baseline phase of large community interventions focusing on modification of cardio-vascular risk factors and conducted in developed countries [19-21].

Despite the refusals and considering that the prevalence rate of *H. pylori *infection was over 50% among individuals non-exposed to most of the exposures investigated, the study had a power of 80% to detect relative risks ≥ 2.0, at the significance level of 5%. Lack of power may have impaired the detection of association between the variables abdominal fullness, early satiety, vomiting, and abdominal bloating, because their prevalence was under 30% in the study population.

Another limitation of this study is the impossibility of declaring causality between associated factors and the outcome. The temporality between exposure and outcome can not be ascertained with precision in cross-sectional studies.

To the authors' knowledge, this is the first population-based study of *H. pylori *infection targeting urban adult population in Brazil. Overall 64.3% of the population ≥ 20 years old was infected with *H. pylori*. This figure is higher than the prevalence of infection detected in population-based studies conducted in developed countries like Australia (30.6%)[19], England (27.6%)[21] and United States (32.7%)[22], and similar to prevalence rates detected in developing settings in South America, Africa and parts of Asia[1]. Considering that the occurrence of infection was significantly associated to dyspepsia even after allowing for confounding and that refusals were more prevalent among non-symptomatic individuals, it is possible that the self-selective inclusion of symptomatic individuals may have in part overestimated the true prevalence of the infection. Projecting the infection rate observed among symptomatic and non-symptomatic complier subjects to symptomatic and non-symptomatic non-complier participants, however, the prevalence rate would be 64.0% (95% CI 60.0%–68.0%).

In the present study past socio-economic variables in childhood presented the strongest association with the occurrence of the infection in adult life (an adjusted increase of approximately 50% in the probability of infection). The age-dependent increase of *H. pylori *prevalence which is observed in most of the studies conducted worldwide lost the significance in the present study when adjusted by socio-economic conditions in childhood. This finding is in agreement with the "birth cohort phenomenon" largely described in the literature. Despite the cross-sectional design and the adverse effect of recall bias, at this and at the study conducted by Mendall *et al*[23] in London, it was possible to explore the association of past socio-economic indicators in childhood with the infection rate in adulthood, a feature generally disregarded in prevalence studies conducted among adult subjects.

The high prevalence rate observed in Pelotas probably reflects infection acquired in childhood and carried on throughout life. In fact, in a population-based study conducted among children from an urban community in north-east Brazil[24], 75.4% were *H. pylori *positive by the age of 12–14 years. Another study conducted among low socio-economic children attending an outpatient clinic in Belo Horizonte, Brazil, showed that infection occurs early and increases with age[25].

Without a known significant animal or environmental reservoir for human strains of *H. pylori*, person-to-person contact appears to be the most likely mode of transmission. As a consequence and as others have shown, the number of siblings, particularly the number of older siblings, domestic crowding and living in orphanages are important determinants of the prevalence of H pylori infection[26,27]. The findings of this study confirmed that larger families and higher exposure as in day-care centres was associated with increased prevalence of the infection.

The study of Replogle *et al*[28] showed a sex difference, higher in men, in the prevalence of infection. The higher prevalence of *H. pylori-*associated diseases like peptic ulcer and gastric cancer in males supports the hypotheses of a real association. In the present study no statistical association was observed between sex and dyspeptic symptoms in crude or in adjusted analysis. However, since the refusal rate was higher among men, it is not possible to exclude with certainty that men with increased risk of infection had not been lost preferably to others. This bias may have masked a real association between sex and *H. pylori *infection, if it actually exists.

Among the demographic variables explored, only ethnicity remained independently associated with the infection. After allowing for present and past socio-economic conditions, non-white individuals presented a probability of infection 32% higher than the observed among the whites. In 1992, Malaty *et al*[29] in a study conducted in the United States with Hispanics matched with blacks and whites for age and socio-economic status, found that the risk of infection was almost identical in Hispanics and blacks and significantly higher than in whites. As suggested by the authors it was probably a reflection of a generation cohort phenomenon related to the generational distance from very low socio-economic status, i.e., the prevalence of *H. pylori *in Hispanics and blacks is currently lower than that of their parents but higher than that of the white population, which has experienced higher socio-economic status for several generations. Lack of information in the present study regarding generational socio-economic conditions impaired to test this hypothesis.

In the study of McQuillan *et al *[22] also conducted in the United States showed that race remained statistically associated with the infection after adjustment for socio-economic factors only in low risk groups, what suggests that ethnicity can be a surrogate for other non-explored factors.

Regarding behavioural factors, the majority of recent studies have not found tobacco use or alcohol consumption to be risk factors for *H. pylori *infection[30]. A study specifically planned to measure whether smoking or consumption of alcohol or coffee was associated with active *H. pylori *in southwest England concluded that smoking or coffee consumption were not related to active *H. pylori *infection and that total alcohol consumption was associated with a small, but not statistically significant, decrease in the odds of infection[31]. In the present study no association was found between these exposures and *H. pylori *infection. These findings however must be seen with caution because changes in lifestyle due to the development of upper gastrointestinal symptoms may mislead the results of cross-sectional studies. In fact, in the present study, changes in alcohol, coffee and mate intakes were reported, respectively, by 35%, 61% and 35% of the participants with dyspeptic symptoms, most of them having reduced the amount or quitted those intakes as an attempt to deal with the symptoms. At the context of the cross-sectional studies, these variables are generally analyzed taking the current status instead of the status before the onset of upper gastrointestinal symptoms as the exposure. A misclassification error moving the association toward the unit is a possible consequence of such approach.

Regarding gastrointestinal complaints only dyspeptic symptoms were significantly associated with the infection. Meta-analyses of trials which have been done in patients with functional (that is, investigated) dyspepsia have shown no benefit from eradication of *H. pylori*[32]. Since individuals classified as presenting dyspeptic complaints in the current study were not investigated for identifying the cause of their symptoms, they may have functional dyspepsia or diseases such as peptic ulcer or gastro-oesophageal reflux disease. At this particular, a randomised placebo controlled trial conducted in 36 family practices in Canada to determine whether a "test for *Helicobacter pylori *and treat" strategy improved symptoms in patients with uninvestigated dyspepsia showed a significant symptomatic benefit at 12 months of follow-up[33].

## Conclusion

In this study, the prevalence rate of *H. pylori *infection was high, following the pattern described by others in developing countries. The socio-economic factors in childhood, the ethnicity and the presence of dyspeptic symptoms were independently associated with the occurrence of the infection.

## Competing interests

The author(s) declare that they have no competing interests.

## Authors' contributions

ISS conceived the study, participated in its design, analysis and coordination, and drafted the manuscript. JB and ASS participated in the design of the study and carried out the isotope analyses. NCJV participated in the design of the study and performed the statistical analysis. CSH, MCB and RDL participated in the design and coordination of the study and helped to draft the manuscript. All authors read and approved the final manuscript.

## Pre-publication history

The pre-publication history for this paper can be accessed here:


